# Ribitol treatment rescues dystroglycanopathy mice with common L276I mutation

**DOI:** 10.1371/journal.pone.0325239

**Published:** 2025-08-20

**Authors:** Bo Wu, Pei Juan Lu, Morgan Drains, Sapana Shah, Anthony Blaeser, Victoria Leroy, Jessalyn Killilee, Molly Holbrook, Qi Long Lu

**Affiliations:** McColl-Lockwood Laboratory for Muscular Dystrophy Research, Atrium Health Musculoskeletal Institute, Carolinas Medical Center, Charlotte, North Carolina, United States of America; Georgetown University Medical Centre, UNITED STATES OF AMERICA

## Abstract

Matriglycan of alpha dystroglycan (α-DG) serves as a receptor for extracellular matrix proteins. Hypoglycosylation of α-DG underlies specific types of muscular dystrophy, dystroglycanopathy. Fukutin Related Protein (*FKRP*) gene encodes a glycosyltransferase that adds ribitol-5-phosphate to the core glycan of α-DG and enables the synthesis of matriglycan. Mutations in the *FKRP* gene are a common cause of dystroglycanopathies. Ribitol is able to restore matriglycan in diseased muscles with FKRP mutations, but this effect relies on partial function of mutant FKRPs. Different mutations affect FKRP function differently, which could affect the efficiency of ribitol treatment. Here we examined the long-term effect of ribitol in mice with *FKRP *C826A (**L276I) mutation, the most common genotype in patient population of LGMD2I/R9. Oral administration of ribitol significantly enhances expression of matriglycan in both cardiac and skeletal muscles up to 40% of normal muscle levels. Importantly, matriglycan is homogeneously expressed in almost all muscle fibers with similar levels especially in cardiac muscle. Consistently, muscle degeneration and regeneration are greatly attenuated with reduced central nucleation and fibrosis especially in the diaphragm. This is associated with improvements in muscle functions, although the diseased mice only show limited deficiency when compared to wild type C57 mice. The higher level of restoration in matriglycan in L276I mice than in P448L mice is consistent with the hypothesis that therapeutic potential of ribitol treatment may depend on the remaining function of mutant FKRPs. These results support clinical trials of ribitol to the majority of patients with FKRP mutations.

## Introduction

The interaction of α-DG with extracellular matrix (ECM) proteins is critical for maintaining muscle integrity [1,2]. This interaction is mediated mainly through the binding of the laminin-binding *O*-mannosylated glycan on α-DG. Recently this glycan has been delineated with the following chain: (3GlcA-β1–3Xyl-α1) *n*-3GlcA-β1–4Xyl-Rbo5P-1Rbo5P-3GalNAc-β1–3GlcNAc-β1–4(P-6) Man-1-Thr/ser [3–5]. This chain extension is completed by the following enzyme activity: POMT1 and POMT2 catalyze the initial *O-*mannosylation of the proteins [6]. The sugar chain is further extended by POMGnT2 (GTDC2) [7,8], B3GALNT2 [9], FKTN, FKRP [3], TMEM5 [10] and B4GAT1 successively [11]. Finally, LARGE produces repeated units of 3GlcA-β1–3Xyl-α1 [12]. Critically, the terminal repeats of 3GlcA-β1–3Xyl-α1 (termed matriglycan) are the actual receptor for binding to ECM proteins. Loss or significant reduction of matriglycan (hypoglycosylation) is the feature of one group of muscular dystrophies known as dystroglycanopathy and caused by mutations in genes involved in matriglycan synthesis and production of dystroglycan [13,14]. Mutations in the *FKRP* gene are among the most common causes of dystroglycanopathy [14–16]. *FKRP* mutations manifest a wide range of disease severity from severe congenital muscular dystrophy (CMD), Walker-Warburg syndrome (WWS), and muscle-eye-brain (MEB) disease to mild limb girdle muscular dystrophy (LGMD) 2I/R9. The variation in phenotypes is related to different locations of point mutations within the coding region of the gene. Homozygotes of the common L276I mutation are generally associated with mild LGMD2I/R9 without the involvement of eye and central nerve system. Compound heterozygotes with one L276I and one other point mutation present more like a mild DMD phenotypes with earlier disease onset. Compound heterozygotes with non-L276I mutations can result in more severe CMD, WWS, and MEB disease [14–16].

The advances in understanding the pathway for *O*-mannosylation of α-DG open new venues for experimental therapy. Specifically, FKRP uses CDP-ribitol as the substrate to add ribitol-5 phosphate (ribitol-5P) to the glycan core on α-DG, thus permitting the synthesis of matriglycan [5,17]. Supplementation of ribitol, a metabolite normally present in the body, can increase the synthesis of CDP-ribitol, which raises the possibility of applying ribitol as a pre-substrate to increase the level of CDP-ribitol and, in-turn, may enhance the efficiency of FKRP as a glycosyltransferase. This therapeutic potential relies on the remaining function of mutant FKRPs [18–20] and has now been demonstrated in mouse model of *FKRP* mutation. Ribitol treatment is able to significantly improve muscle pathology and functions in the *FKRP* mutant mice with P448L mutation which is associated with CMD in clinic [21,22]. Moreover, long-term use of ribitol, shows no detectable side effects in muscle development, body weight, behavior of the animals and in histology and functions of vital organs including liver and kidney. Results from phases I and II clinical trials are also encouraging [ClinicalTrials.gov Identifier: NCT04800874 and NCT05775848].

Different *FKRP* point mutations affect the function of the glycosyltransferase differently [23,24]. This raises the question of whether therapeutic effect of ribitol could be mutation-type dependent. Since the therapeutic effect of ribitol relies on the remaining function of mutant FKRPs, it is possible that mutations such as the common L276I mutation, which retains better function of the gene, might be more effective in utilizing the increased substrate for matriglycan synthesis. In this study, we examined the effect of ribitol in the mouse model with homozygous L276I mutation. Despite the mild phenotype of the L276I homozygotes, results from this study clearly show that ribitol treatment provides therapeutic benefit. Matriglycan expression is significantly enhanced and associated with improvement in skeletal muscle function. Muscle pathology is also improved with reduced fibrosis and central nucleation. These results confirm the efficacy of the treatment to the diseases with common L276I mutation and further support the wider applicability of ribitol treatment to other FKRP mutations.

## Materials and methods

### Animal care

Animal studies were approved by the Institutional Animal Care and Use Committee (IACUC), Atrium Health. All mice were housed in the vivarium of Atrium Health following guidelines of the institute. Food and water were given *ad libitum* during all phases of the study Animals were ear tagged prior to group assignment. Body weight was measured daily for minoring mouse condition and for accurate dosing by gavage.

### Mouse model and experimental procedure

**Fig 1:**
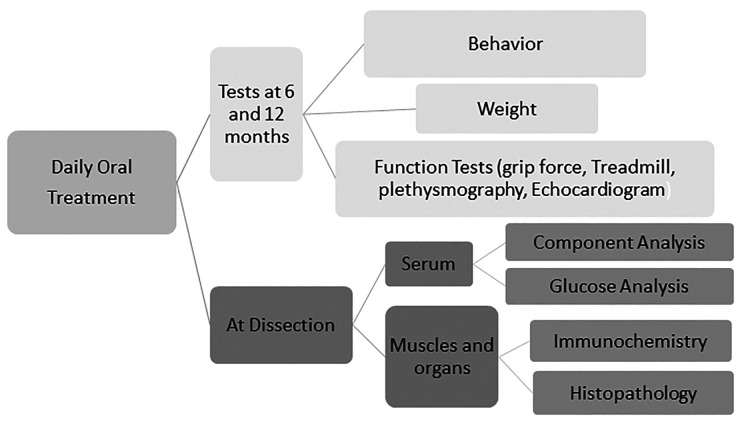
Diagramatic illustration of the animal procedures.

FKRP-L276I (L276I) mutant mice (C57BL/6N background) containing a homozygous missense mutation (c.826C > A, p.Leu276Ile) were reported earlier [23,24]. C57BL/6 mice were purchased from Jackson Laboratory. Ribitol was from Sigma (A5502 Adonitol, ≥ 98%, Sigma, St. Louis) and dissolved in saline for gavage as 100 μl/10g mouse body weight. Nine week old mice were treated with ribitol by gavage with the following doses and regimes: 0.5g/kg, 2g/kg, 5g/kg body weight daily. Ten mice (5 mice for each sex) were randomly assigned to each groups. Age-matched L276I mice were gavaged with the same volume of saline as controls. Regular muscle function was tested and euthanization was carried out by cervical spine dislocation under isoflurane anesthesia after 12 months of treatment and tissues including liver, spleen, kidney, heart, diaphragm, TA, quadriceps were collected for analyses (The procedures are illustrated in Fig 1).

### Immunohistochemical and western blot analysis

Tissues were dissected and snap-frozen in dry-ice-chilled-2-methylbutane. Immunohistochemical detection of matriglycan was carried out as described previously with matriglycan recognized by antibody IIH6C4 (EMD Millipore) (1:500) [22]. AlexaFluor 488 or 594 goat anti-mouse IgM (Invitrogen) (1:500) were used for visualization. Slides were examined in a blind manner by the investigators using an Olympus BX51/BX52 fluorescence microscope (Opelco) and images were captured using the Olympus DP70 digital camera system (Opelco).

Western blot (WB) analysis was carried out with the method described previously [22]. Briefly, fifty μg of protein was loaded on an 4–15% Bio-Rad Mini-PROTEAN TGX gel (Bio-Rad) and immunoblotted. Half of the amount of total protein was loaded for muscle from C57 control mice. Nitrocellulose membranes were incubated with primary antibodies IIH6C4 (1:1000), and α-actin (Sigma) (1:2000) overnight at 4°C. Horseradish peroxidase (HRP)-conjugated secondary antibodies were incubated for 2 hr at room temperature and the blots were developed by electrochemiluminescence immunodetection (PerkinElmer). ImageJ software was used for quantification from the blot.

### Histopathological and morphometric analysis

Sections were stained with hematoxylin and eosin (H&E) and Masson’s Trichrome. Cross-sectional fiber diameter were determined from tibialis anterior (TA) and quadriceps stained with H&E using MetaMorph v7.7 Software (Molecular Devices). Percentage of centrally nucleated fibers (CNF) were manually quantified from the same tissue sections stained with H&E. Fibrotic area in the Masson’s Trichrome stained sections was quantified from heart, diaphragm, and TA using ImageJ software. A total of 500 fibers from five representative 20X magnification images per each muscle per animal were counted for all the morphometric analyses. Fiber membrane signal intensity was measured by ImageJ Point Tool as described before [25].

### Grip strength and treadmill test

Grip strength was assessed using a grip strength meter from Columbus Instruments, Columbus, OH. Five successful forelimb and hindlimb strength was measured within 2 minutes, and data were normalized to body weight. Treadmill test was performed as described in the previous study [22]. LE8700 treadmill (Panlab/Harvard Apparatus, Barcelona, Spain) supplied with shock grids mounted at the back of the treadmill was used to deliver a 0.2 mA current to provide motivation for exercise. Mice were run until exhaustion demonstrated by the animal remaining on the shock grid for 10 consecutive seconds without getting off or 50% on/off within 1-minute period [22,23].

### Whole body plethysmography

Respiratory functional analysis in conscious, freely moving mice were performed using a whole-body plethysmography technique described previously [22]. Constant air flow of the plethysmograph apparatus (Emka Technologies, Falls Church, VA) was maintained through the connection to a ventilation pump. A computer running EMKA iox2 software with the respiratory flow analyzer module was used to detect pressure changes due to breathing and recording the transducer signal. Mice were placed inside the “free moving” plethysmograph chamber and allowed to acclimate for 5 min to minimize any effects of stress related changes in ventilation. Resting ventilation was measured for a duration of 15 min after the acclimation period. Body temperatures of mice were maintained to be constant at 37°C during the ventilation protocol.

### Echocardiogram

Echocardiogram was performed using the BioscanSonixTablet Ultrasound System (Analogic Ultrasound, Peabody, MA). Detailed method was published by Blaeser et al. [23].

### Statistical analysis

Results were expressed as means + SEM. One-Way ANOVA was performed for comparing treatments with control group. Statistical significance was set at *p ≤ 0.05* (*).

## Results

### One year ribitol treatment enhances matriglycan expression in both cardiac and skeletal muscles

**Fig 2:**
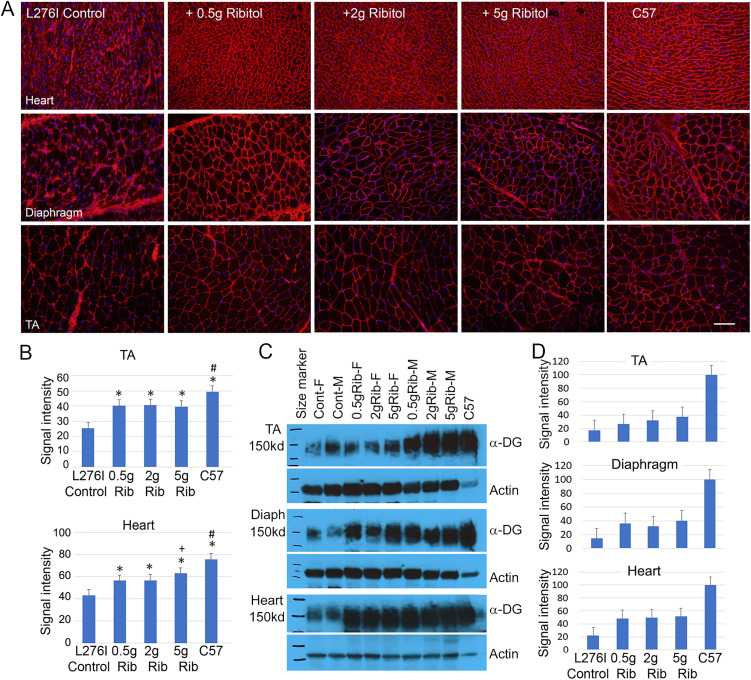
Induction of matriglycan in cardiac and skeletal muscles by ribitol. (A) Immunohistochemical staining with IIH6C4 antibody of heart, diaphragm and tibialis anterior (TA) muscles from the control and ribitol-treated mice (left and middle 3 panels, respectively) and C57 mice (right panel). Control (Cont) mice were given saline only. Scale bar, 100 µm. Cellular nuclei were counterstained with DAPI (blue). (B) Signal intensity measurement by ImageJ Point Tool. 0.5g Rib, 0.5g/kg ribitol treatment; 2g Rib, 2g/kg ribitol treatment; 5g Rib, 5g/kg ribitol treatment. *p ≤ 0.05 when compared to L276I controls. # significant when compared to all the ribitol-treated groups. + , when compared to the two low dose ribitol treatments. (C) WB analysis of protein lysates from heart, TA and diaphragm (diaph) of two saline-treated (-) and two each of the 0.5g, 2g and 5g/kg daily ribitol-treated L276I mice. F, female mice; M, male mice. *C57* mice were used as positive control. Matriglycan was detected by blotting with IIH6C4 antibody and detection of α-actin was used as loading control. (D) Signal intensity measurement with ImageJ. Values of the 2 samples of same treatment were normalized to α-actin expression for each tissue and presented as percentage expression compared to C57. Error bars represent mean + SEM. One-Way ANOVA was used for comparing treatments with control group *p ≤ 0.05 in comparison with saline-treated controls.

FKRP-L276I homozygous mice show delayed onset of disease phenotype, with muscle degeneration starting at about 2 months of age [26]. We therefore chose to treat the mice starting at 2 months of age with daily gavage of ribitol at 0.5g/kg, 2g/kg, and 5g/kg of body weight. The L276I mutation causes significant reduction in matriglycan expression [26]. However, matriglycan can still be clearly detected with IIH6 antibody in both cardiac muscle and skeletal muscles including diaphragm. As shown in Fig 2A, weak and partly irregular membrane staining of matriglycan was detected in the majority of muscle fibers. The progressive muscle degeneration in the control muscles was also indicated by the background staining appearing as irregular speckles and thick lines between muscle fibers. This is in contrast to strong and continuous membrane staining without speckles of the same tissues in C57 control muscles. Ribitol treatment with all 3 doses clearly improved the signal intensity of matriglycan with more homogenous distribution along the entire membrane of almost all muscle fibers. Similar to the wild type C57 muscles, background staining of irregular speckles and thick lines between fibers was almost absent in all muscles from the treated mice. Signal intensity measured by ImageJ Point tool showed a dose-related enhancement (Fig 2B). Importantly, all three muscle types showed significant enhancement in matriglycan levels in all 3 dose groups when compared to the saline treated control. Enhancement in matriglycan expression was also confirmed by WB (Fig 2C and D). The levels of matriglycan in the ribitol-treated groups almost doubled the amount of the saline-treated control with the heart of the highest dose group reaching more than 40% of wild type levels. Similar levels of enhancement were detected in both male and female mice treated by ribitol (Supplementary Fig. 1 A and B in S1 File).

### Ribitol treatment improves histopathology

**Fig 3:**
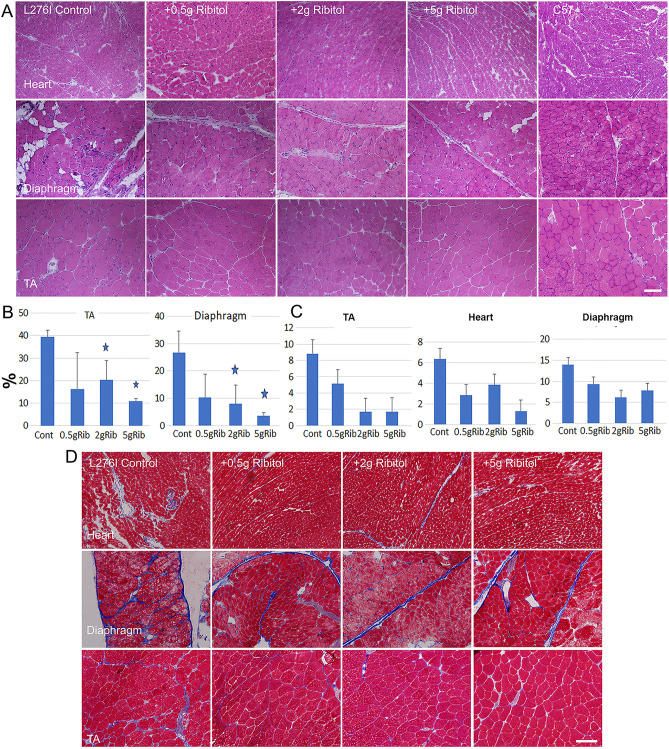
Effect of one year ribitol treatment on muscle pathology. (A) Histology of heart, diaphragm and tibialis anterior (TA) muscles with H&E staining. (B) Analysis of central nucleation in TA and diaphragm (percent of total fibers), and (C) percentage of fibrosis in TA, heart and diaphragm. (D) Masson Trichrome staining for measurement of fibrosis (blue areas represent fibrotic tissues). 0.5gRib, 0.5g/kg ribitol treatment; 2gRib, 2g/kg ribitol treatment; 5gRib, 5g/kg ribitol treatment. Error bars represent mean + SEM. One-Way ANOVA was used for comparing treatments with control group. *p ≤ 0.05 in comparison with saline-treated control. Scale bar, 100 µm.

At the age of 14 months, when the saline treated L276I mice were examined, TA muscles showed significant variation in fiber size and the presence of a high percentage of CNF reaching almost 40% (Fig 3). The most conspicuous changes were in the diaphragm showing wide-spread focal degeneration and regeneration with variable fiber size and central nucleation. Infiltration of both fat and fibrotic tissues became obvious although muscle fibers remained as the dominant tissue component. Treatment with ribitol improved pathology in both skeletal muscles with rare focal degeneration and reduction of fat and fibrotic tissues. Consistently, the percentage of CNF was reduced in all three treated groups to less than 10% in the group of the highest dose of ribitol. No obvious pathology was observed in the cardiac muscle. However, improvement in histology can still be detected by measuring the fibrotic areas with Masson Trichrome staining, especially with 2g/kg and 5g/kg ribitol treatments (Fig 3C & D).

### Low doses of ribitol treatment improve muscle function

**Fig 4:**
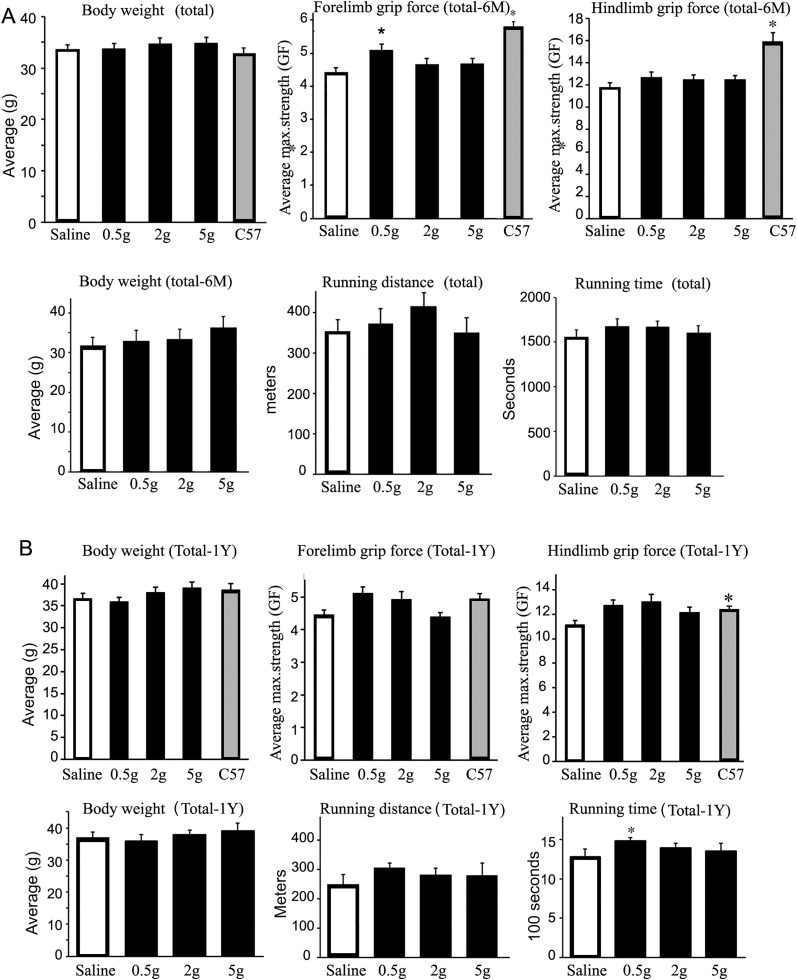
Effect of ribitol treatment on muscle function by grip force measurement and treadmill exercise. Mice were treated from 9 weeks of age. Control mice were given saline only. Body weight, grip force measurement and treadmill exercise at 6 months of treatment (A) and 12 months of treatment (B). Improvement in functions are observed with significance for forelimb grip force in the 0.5g/kg treated mice at 6 months and for running time for the 0.5g/kg ribitol treatment group. Treadmill exhaustion test assessing distance (m, meters) and running time (min, minutes) in control, ribitol-treated L276I mutant mice and *C57* mice (n = 10). Gram Force (gf) is normalized to bodyweight (g) for final gf/g. 0.5g, 0.5g/kg ribitol treatment; 2g, 2g/kg ribitol treatment; 5g, 5g/kg ribitol treatment. Error bars represent mean + SEM. One-Way ANOVA was used for comparing treatments with control group. *p ≤ 0.05 is considered statistically significant.

Effects of ribitol treatment on the general health of the mice was monitored through body weight measurement once every 2 weeks as well as daily cage-side observation, according to the Atrium Health IACUC approved protocol. Skeletal muscle function was assessed at 6-month and 12-months post-treatment. There was a slight increase in body weight for all mice after 6-months of treatment. However, when separated by sex, the treated males showed a clear increase whereas there was no increase in the treated female mice (Fig 4 and Supplementary Fig. 2 in S1 File). Body weight for both sexes at 12 months was higher in most of the treated groups compared to the control, but without statistical significance. Grip force measurement showed a trend of improvement in both forelimb and hindlimb with the 0.5g/kg treated group reaching significance (Fig 4A). Improvement in grip force was maintained after 12-month treatment, but without significance when both males and females were analyzed together (Fig 4B). However, when analyzed separately, females improved more than males (Supplementary Fig. 2 in S1 File), possibly related to the fact that the treated male mice gained more weight when compared to the saline-treated male controls. The degree of improvement in treadmill exercise was similar for all of the ribitol-treated groups at 12-months with the 0.5g/kg group being statistically significant when compared to saline-treated controls. The changes were observed in both treated male and female groups (Supplementary Fig. 3 in S1 File). The relatively muted improvement in muscle functions in the 5g/kg group is likely related to discomfort of the high dose treatment, which leads to reduced activity and higher body weight. This, together with the limited pathology of the diseased mice and size of each cohort, are the likely causes for the lack of a clear dose dependency in muscle function by the two tests

### Ribitol treatment improves cardiac and respiratory functions

**Fig 5:**
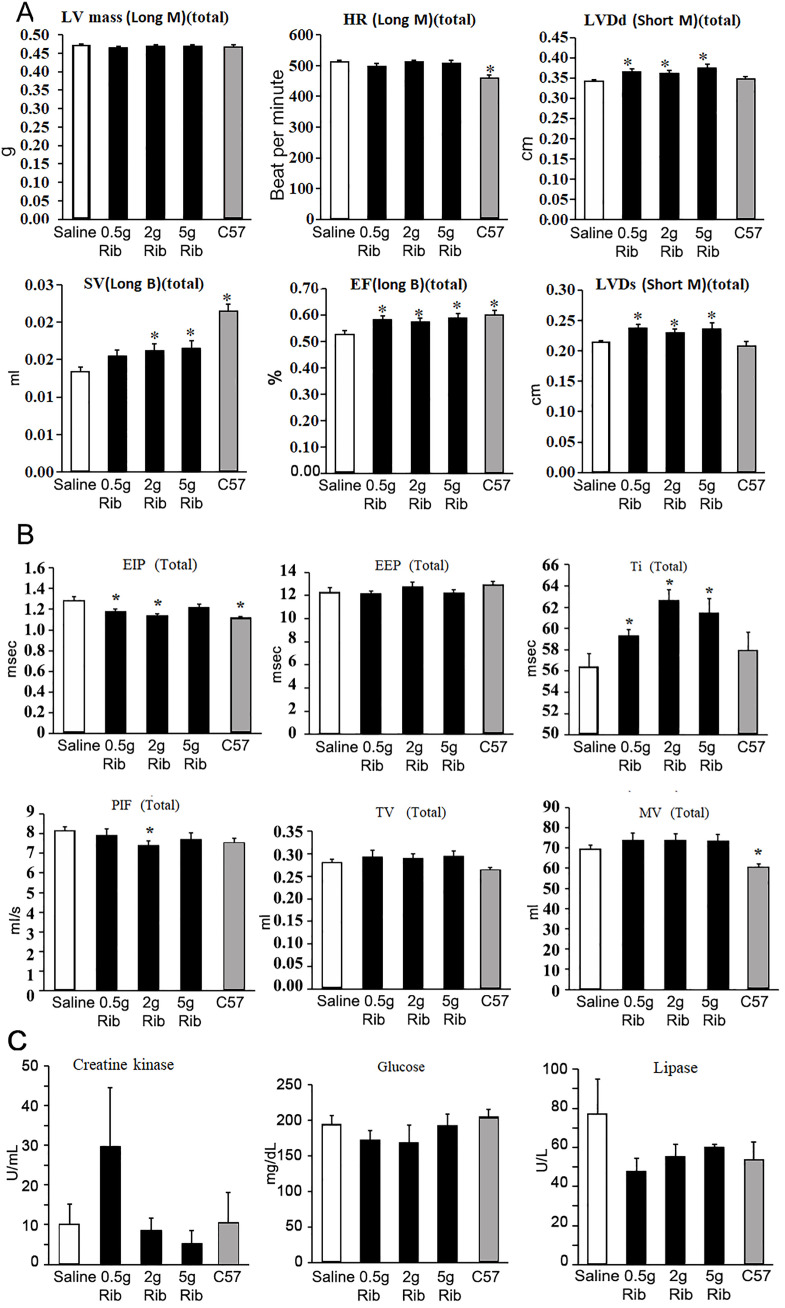
Effect of ribitol on cardiac and respiratory functions and serum markers. (A) Echocardiography. Abbreviations: LV, Left ventricle; HR, Heart rate; SV, stroke volume; EF, ejection fraction; LVDd and LVDs, left ventricle diameter in diastole and systole respectively. (B) Plethysmography. Abbreviations: EIP, end-inspiratory pause; EEP, end-expiratory pause; Ti, inspiratory time; PIF, peak inspiratory flow; TV, tidal volume; MV, minute volume. (C) Serum markers. 0.5gRib, 0.5g/kg ribitol treatment; 2gRib, 2g/kg ribitol treatment; 5gRib, 5g/kg ribitol treatment. Error bars represent mean + SEM. One-Way ANOVA was used for comparing treatments with control group. *p ≤ 0.05 is considered statistically significant.

Focal and scattered fibrosis was observed in the cardiac muscle of the homozygous L276I mice [23,24]. Although mild, cardiac muscle of the mutant mice displays detectable contractile dysfunction by the age of 7 months with cardiography in parameters such as ejection fraction (EF) and fractional shortening (FS) [26]. Consistently, the saline-treated L276I mice at the age of 14 months displayed significant deficiencies in stroke volume (SV) and EF (Fig 5A). One year of ribitol treatment greatly improved SV with significance in the two higher dose groups. Similarly, significant improvement was detected for EF in all three dose groups. These changes were associated with a significant increase in left ventricle internal diameter both in diastole (LVDd) and systole (LVDs). There was a slight reduction in heart rate (HR) trending towards wild type C57 mice, but no change in left ventricle (LV) mass. End-diastolic volume was also slightly increased trending towards wild type C57 mice (Supplementary Fig. 4 in S1 File).

The mild fibrosis in the diaphragm results in limited respiratory dysfunction of the saline-treated L276I mouse [22–24,26]. The most consistent deficiency detected in FKRP mutant mice using the non-invasive whole-body plethysmography is the increased time in end-inspiratory pause (EIP) and peak inspiratory flow (PIF) (Fig 5B). Ribitol treatment reduced the time of these two parameters with significance detected in both lower dose groups. In contrast, ribitol treatment increased tidal volume (TV), minute volume (MV) and especially inspiratory time (Ti) with statistical significance. Furthermore, apparent reduction in the expiratory time (Te) and relaxation time (RT), but an increase in enhanced pause (Penh) were noted in the ribitol-treated groups. No apparent changes were detected in several other respiratory parameters (Supplementary Fig. 5 in S1 File).

### Ribitol treatment has no significant effect on creatine kinase, but lowers glucose levels in serum

Ribitol treatment showed limited effect on serum markers of the L276I mutant mice. There was no significant changes in levels of creatine kinase (CK) of the treated animals when compared to both saline-treated L276I and wild type C57 mice, although the 5g/kg ribitol treatment group displayed the lowest levels (Fig 5C). This is likely related to the limited pathology and old age (14 months) (less active) of the mice when the serum samples were obtained. The generally lower levels of glucose in the treated groups than in the saline controls are interesting as it shows that the long-term use of therapeutic amounts of ribitol does not cause hyperglycemia, a concern for a therapy with a sugar-related product. Also interesting is the apparent decreased levels of lipase in all of the ribitol-treated groups and significantly higher serum calcium levels in the 5g/kg ribitol-treated group when compared to that in the saline-treated mice. The significance of these changes are not yet understood. No clear changes were detected in other markers (Supplementary Fig. 6 in S1 File).

## Discussion

The most important biomarker for dystroglycanopathy is the matriglycan which serves as a receptor for the linkage between the cell membrane and ECM proteins, loss of which is the direct cause for all dystroglycanopathy [27,28]. The levels of matriglycan expression therefore should be directly related to disease severity and progression as discussed in a recent review [25]. Results from the earlier reports suggest that less than 10% of normal levels of matriglycan is produced in the P448L mutant mice with severe disease phenotype [16,22,29]. Such low levels of matriglycan are difficult to detect by WB with currently available methods. Muscles from the L276I mutant mouse have about 20% of normal matriglycan which can be consistently detected. Ribitol treatment almost doubles the amount of matriglycan in muscles of the L276I mouse, greatly diminishing the pathology even in the most severely affected diaphragm. These results together reinforce the direct correlation between levels of matriglycan and disease severity. However, the levels of matriglycan required to achieve significant efficacy are yet to be determined and likely dependent on the endogenous levels of matriglycan in the diseased muscles as well as disease stage before treatment. It is worth noting that different treatments may result in different patterns of enhancement in matriglycan expression within muscle tissues. Long-term ribitol treatment produced highly homogenous enhancement to almost all muscle fibers of both L276I and P448L mutant mice. This distribution pattern of matriglycan would likely result in improvement in disease phenotype as achieved by similar levels of endogenous matriglycan. In contrast, adeno-associated virus (AAV) gene therapy so far produces highly variable levels of matriglycan even within a single muscle tissue, which may well provide lower efficacy than the same levels of homogenous matriglycan expression, especially in the long-term [30,31].

Despite the late onset and mild disease phenotype, the L276I mutation is frequently associated with dilated cardiomyopathy and ventilatory impairment [32–34]. Importantly, both heterozygotes and homozygotes for the common mutation can develop cardiac dysfunction and failure. Further, progressive muscle degeneration and fibrosis in the diaphragm are much greater than any other types of muscles, although such a selective vulnerability of respiratory muscles is not understood. This often leads to respiratory failure which can precede the onset of profound weakness in other skeletal muscles. Therefore, improvement in pathology and function of these two organs are perhaps more important than improvement to skeletal muscles in general. Similar to the phenotypes in patients and despite the later onset and mild muscle pathology, the homozygous L276I mutant mice display pathology and dysfunction in both organs at the age of 14 months. Encouragingly, ribitol treatment achieves clearly detectable improvements in pathology and functions of both organs. EF and SV are the commonly used indexes of left ventricle contractile function and represent the fraction of blood sent from the left and right ventricle with each heartbeat [26]. All three doses improve the EF and SV respectively, with the two higher dose treatments showing statistical significance. Ribitol treatment also improves pathology and respiratory function, specifically EIP and PIF with significance. These results clearly indicate the potential of ribitol treatment for protection of these organs from severe dysfunction and failure.

## Conclusion

This study, consistent with our early studies in the P448L FKRP mutant mice, further demonstrates significant efficacy of ribitol treatment for the most common L276I mutation of FKRP. Despite the later disease onset and mild functional defect in the mutant mice, long-term ribitol treatment improves all aspects of the disease phenotype without detectable side effects. Significant improvement in muscle pathology and functions, especially in cardiac and respiratory functions, supports application of ribitol treatment for a wide genetic spectrum of patients with FKRP mutations.

## Supporting information

S1 File Supplementary Figure 1 – figure 6. Original WB blots. (PDF)
