# Supplementary material for: Ribitol treatment rescues dystroglycanopathy mice with common L276I mutation
Source: PLoS One. 2025 Aug 20;20(8):e0325239. doi: 10.1371/journal.pone.0325239 (PMC12367137; doi:10.1371/journal.pone.0325239)
Supplement: S1 File — (PDF) [file pone.0325239.s001.pdf]

## <u>Supplementary figure 1 – figure 6</u>

A

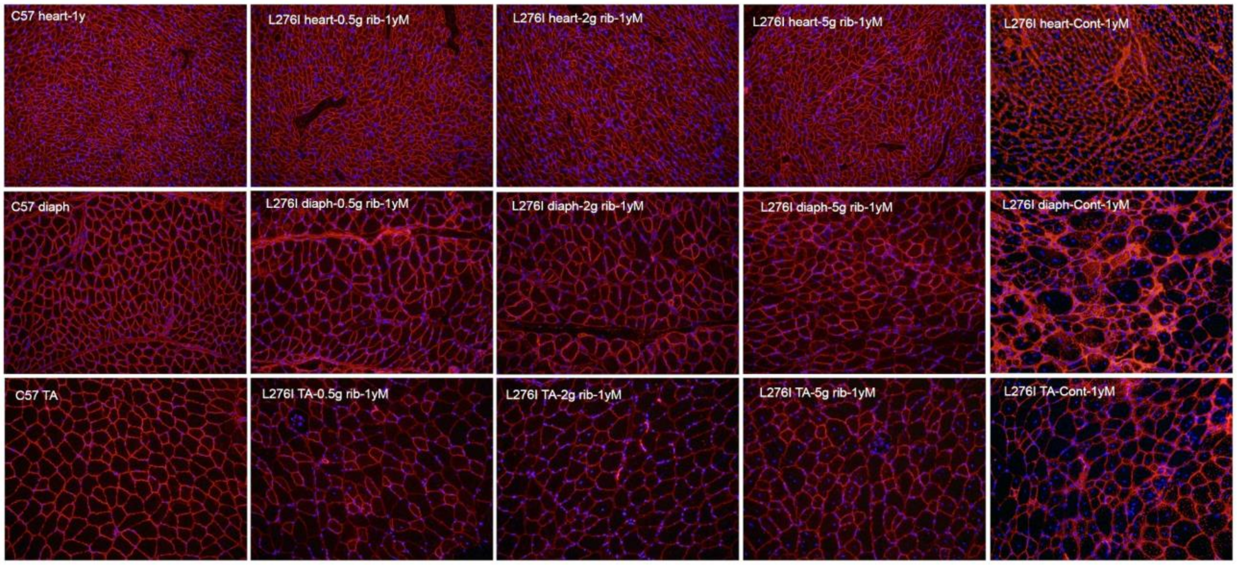


B

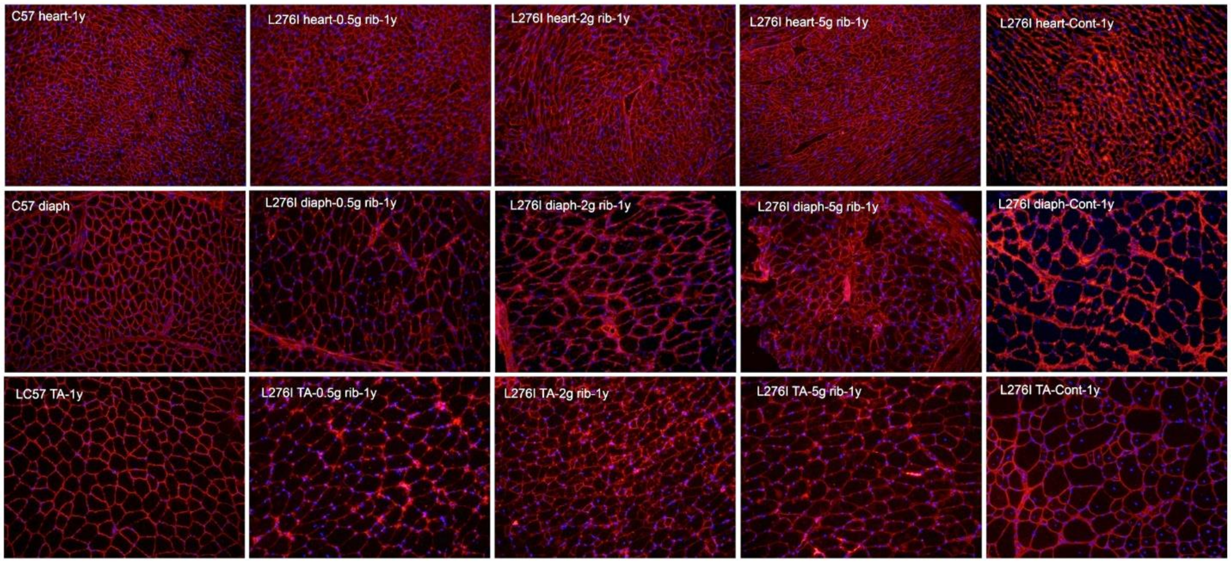


**Supplementary Figure 1. Induction of matriglycan in cardiac and skeletal muscles in male (M) (A) and female (B) mice after one year ribitol treatment.** Mice were treated from 9 weeks of age. Control mice were gavaged with saline only. Immunohistochemical staining with IIH6C4 antibody of heart, diaphragm (diaph) and tibialis anterior (TA) muscles from the control C57 mice (left panel), ribitol-treated mice and saline-treated control (Cont) L276I mice (middle 3 and

right panels, respectively). Nuclei were counterstained with DAPI (blue). 1y, one year treatment. 0.5g rib, 2g rib and 5g rib are mice treated with o.5g/kg, 2g/kg, and 5g/kg ribitol daily respectively.

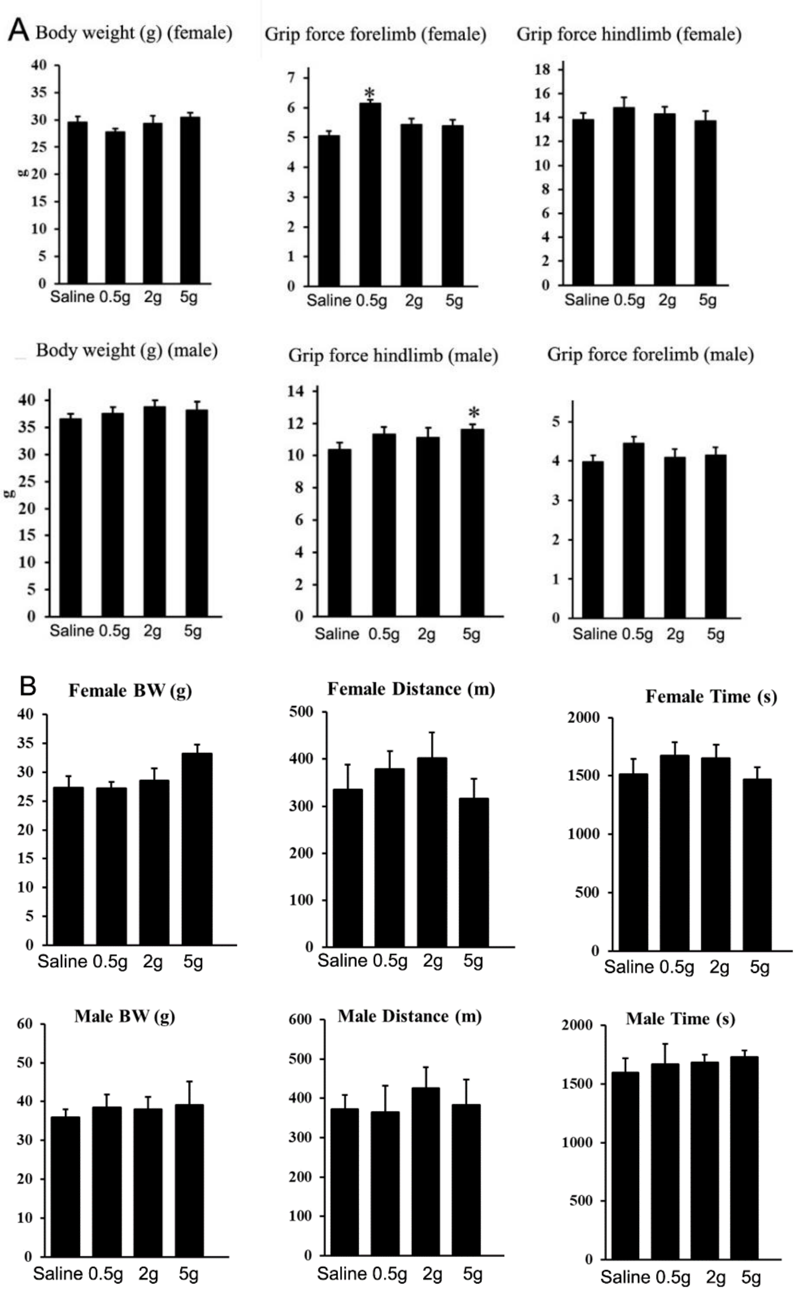

**Supplementary Figure 2. Effect of 6-month ribitol treatment on muscle function of male and female L276I mice by grip force measurement (A) and treadmill exercise (B).** Mice were treated from 9 weeks of age. Control mice were gavaged with saline only. Assessing distance (m, meters) and running time (min, minutes) in saline-treated and ribitol-treated mutant mice (n=5). Mice were treated with 0.5g, 2g, and 5g ribitol per kg bodyweight (BW) daily respectively. Grip force is normalized to bodyweight (g). G, gram; m, meter(s); s, second(s). *$p \leq 0.05$ is considered statistically significant. Error bars represent mean ± SEM. One-Way ANOVA was used for comparing treatments with control group.

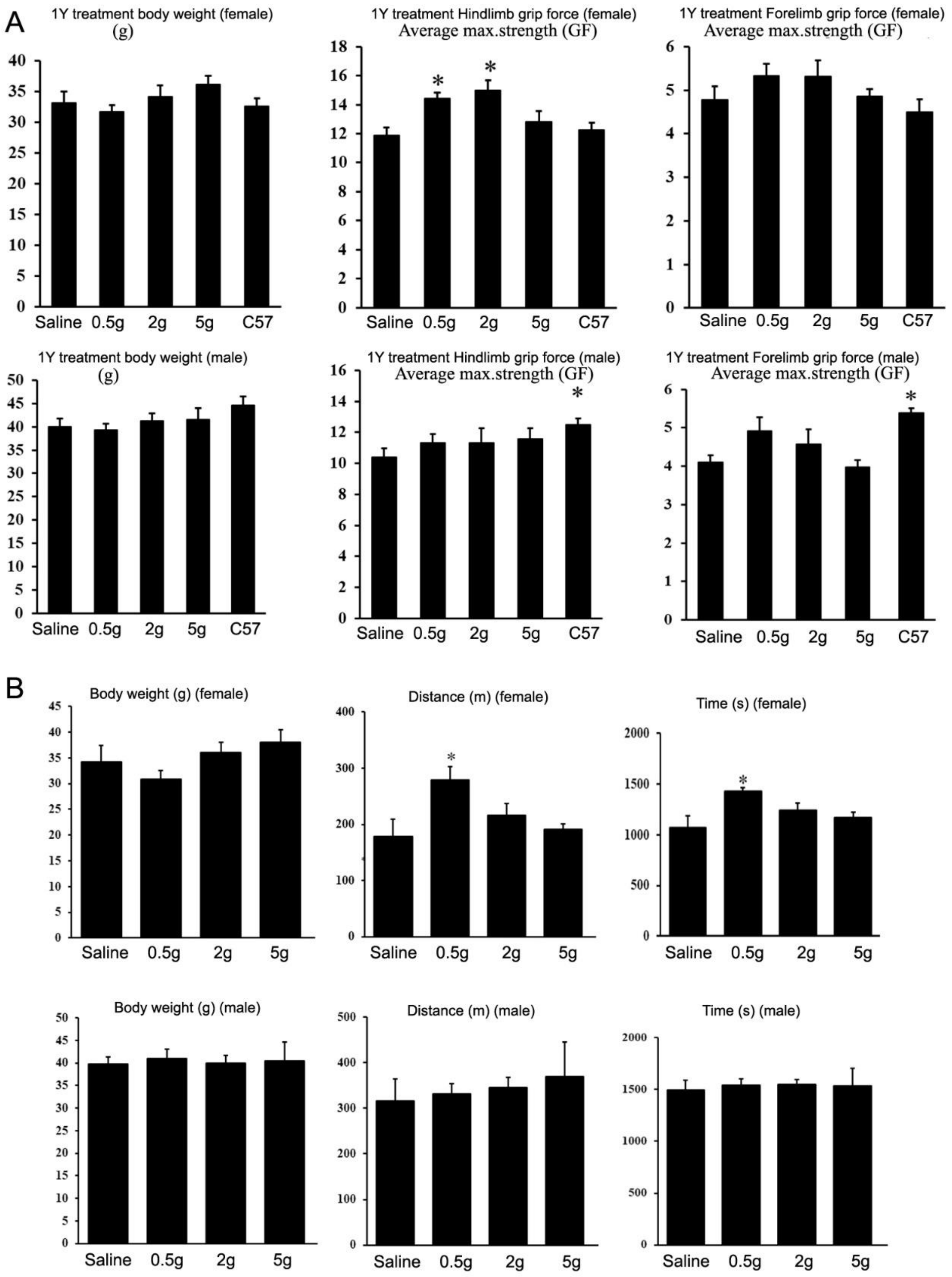


**Supplementary Figure 3. Effect of 1 year ribitol treatment on muscle function of male and female L276I mice by grip fore measurement (A) and treadmill exercise (B).** Mice were

treated from 9 weeks of age with 0.5g, 2g, and 5g ribitol per kg bodyweight daily respectively. Saline-treated mice were used as control (n=5). Grip force (GF) is normalized to bodyweight (g, Y axis). Running distance is measured in meters (m) and running time in seconds (s). *$p<0.05$ is considered statistically significant. Error bars represent mean ± SEM. One-Way ANOVA was used for comparing treatments with control group.

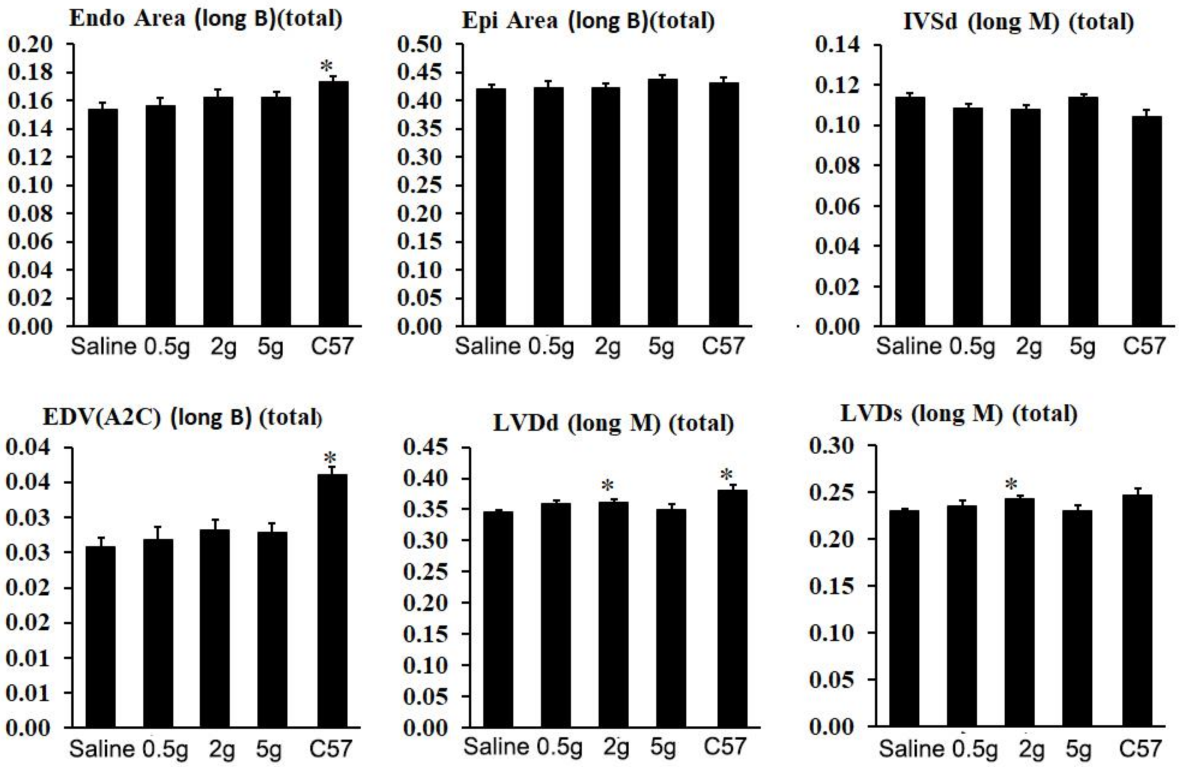


**Supplementary Figure 4**. **Effect of ribitol on cardiac functions of the L276I mutant mice examined by echocardiography**. Mice were treated from 9 weeks of age for 1 year with 0.5g/kg, 2g/kg and 5g/kg bodyweight ribitol. Abbreviations: Endo and Epi, endocardium and epicardium; IVsd, Interventricular septal diastole; EDV, End diastolic volume; LVDd and LVDs, Left ventricle diameter in diastole and systole respectively. Error bars represent mean ± SEM. One-Way ANOVA was used for comparing treatments with control group. *$p \leq 0.05$ is considered statistically significant.

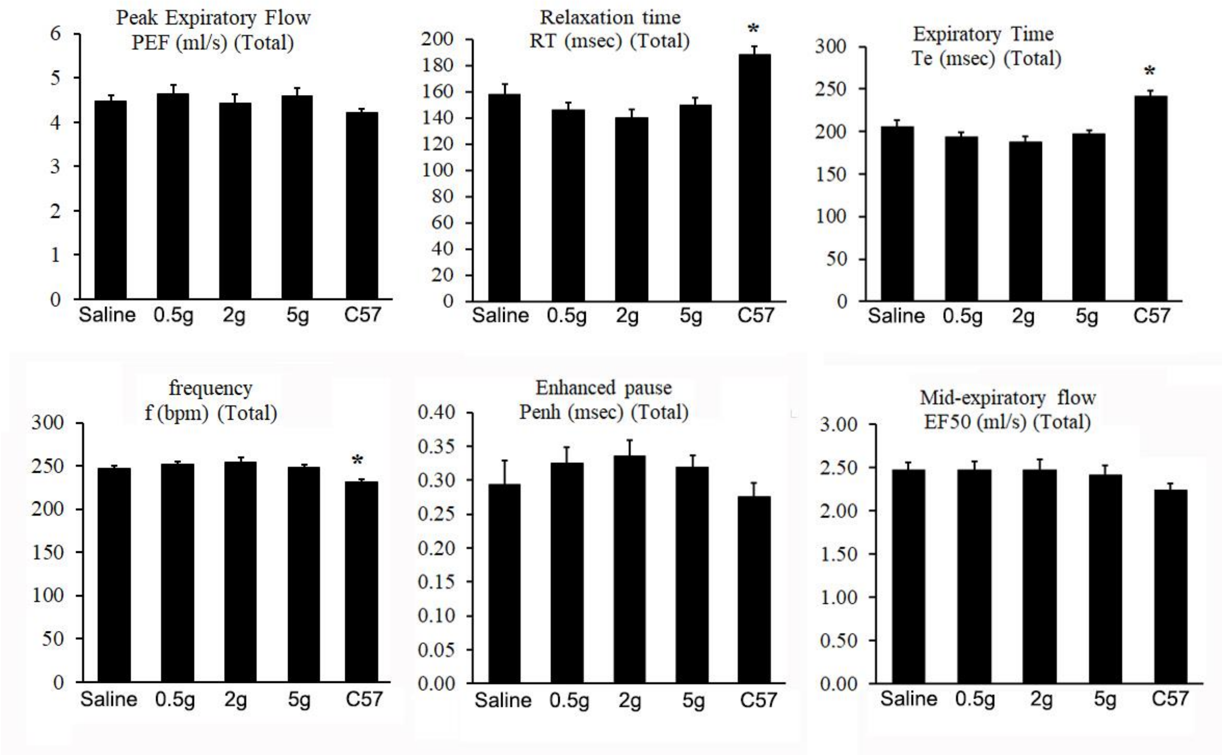


**Supplementary Figure 5. Effect of ribitol on respiratory functions of the L276I mutant mice examined by Plethysmography.** Mice were treated from the age of 9 weeks for 1 year with 0.5g/kg, 2g/kg and 5g/kg bodyweight ribitol. Saline treated mice and untreated C57 mice were used as control. S, second; msec, millisecond; bpm, breath per minute. Error bars represent mean ± SEM. One-Way ANOVA was used for comparing treatments with control groups. *$p \leq 0.05$ is considered statistically significant.

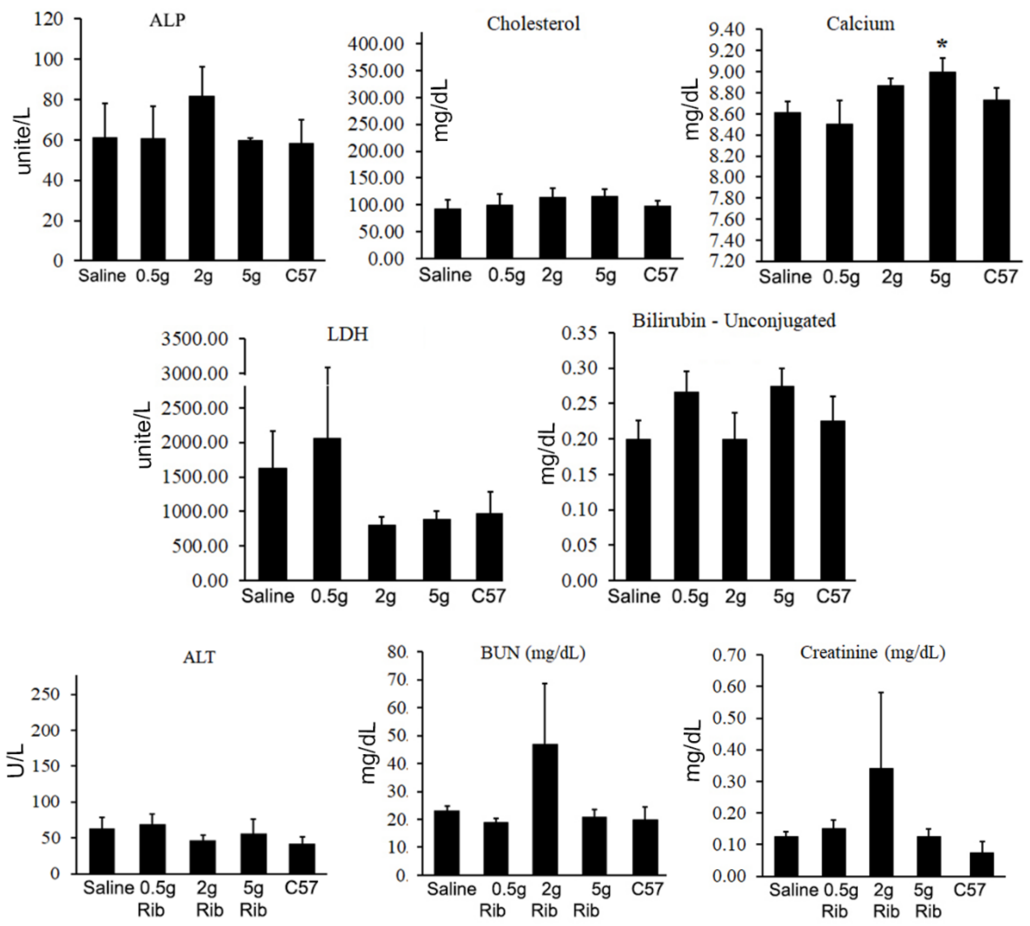


**Supplementary Figure 6. Serum marker detection in ribitol treated L276I mice**. ALP, alkaline phosphatase. LDH, lactate dehydrogenase. Mice were treated from 9 weeks of age for 1 year with 0.5g/kg, 2g/kg and 5g/kg bodyweight ribitol. Saline treated mice and untreated C57 mice were used as control. U/L, Unit per liter; dL, deciliter. One-Way ANOVA was used for comparing treatments with control groups. Error bars represent mean +SEM test. *$p \leq 0.01$. Error bars represent mean + SEM. One-Way ANOVA was used for comparing treatments with control group. *$p \leq 0.05$ is considered statistically significant.
